# *Sleeping Beauty* Mouse Models Identify Candidate Genes Involved in Gliomagenesis

**DOI:** 10.1371/journal.pone.0113489

**Published:** 2014-11-25

**Authors:** Irina Vyazunova, Vilena I. Maklakova, Samuel Berman, Ishani De, Megan D. Steffen, Won Hong, Hayley Lincoln, A. Sorana Morrissy, Michael D. Taylor, Keiko Akagi, Cameron W. Brennan, Fausto J. Rodriguez, Lara S. Collier

**Affiliations:** 1 School of Pharmacy and University of Wisconsin Carbone Cancer Center, University of Wisconsin, Madison, Madison, WI, United States of America; 2 Human Oncology and Pathogenesis Program, Memorial Sloan-Kettering Cancer Center, New York, New York, United States of America; 3 Division of Neurosurgery, Arthur & Sonia Labatt Brain Tumour Research Centre, The Hospital for Sick Children, Toronto, ON, Canada; 4 Comprehensive Cancer Center, The Ohio State University, Columbus, OH, United States of America; 5 Department of Pathology, Division of Neuropathology, Johns Hopkins University, Baltimore, MD, United States of America; Southern Illinois University School of Medicine, United States of America

## Abstract

Genomic studies of human high-grade gliomas have discovered known and candidate tumor drivers. Studies in both cell culture and mouse models have complemented these approaches and have identified additional genes and processes important for gliomagenesis. Previously, we found that mobilization of *Sleeping Beauty* transposons in mice ubiquitously throughout the body from the *Rosa26* locus led to gliomagenesis with low penetrance. Here we report the characterization of mice in which transposons are mobilized in the Glial Fibrillary Acidic Protein (GFAP) compartment. Glioma formation in these mice did not occur on an otherwise wild-type genetic background, but rare gliomas were observed when mobilization occurred in a *p19Arf* heterozygous background. Through cloning insertions from additional gliomas generated by transposon mobilization in the *Rosa26* compartment, several candidate glioma genes were identified. Comparisons to genetic, epigenetic and mRNA expression data from human gliomas implicates several of these genes as tumor suppressor genes and oncogenes in human glioblastoma.

## Introduction

High-grade gliomas are aggressive and invasive primary brain tumors with limited treatment options and a poor prognosis. The debilitating nature of the illness and poor results of therapy led to high-throughput genomic studies of human high-grade gliomas by The Cancer Genome Atlas (TCGA) and others [1], [2]. Genetic studies in mouse models serve as a complementary approach to human tumor genomic efforts to identify driver genes involved in glioma formation. Our previous studies [3], [4] demonstrated that the *Sleeping Beauty* (SB) transposon technology is capable of generating gliomas due to insertional mutagenesis of glioma genes. In these studies T2/onc transposons were mobilized from low-copy (LC, approximately 25 copies) concatemers throughout the body due to expression of SB transposase from the *ROSA26* locus (Rosa26-SB11). Although approximately 90% of Rosa26-SB11; T2/onc LC mice developed leukemia, 14% of these mice harbored gliomas, primarily anaplastic astrocytomas. Tumor-predisposed genetic backgrounds increased glioma formation in mice with mobilizing transposons. Cloning insertions from these SB-induced gliomas identified *Sfi1*, *Csf1*, *Mkln1*, *Vps13a* and *Fli1* as common insertion sites (CISs) which are chromosomal regions that are insertionally mutated in more tumors than would be expected by random chance and represent candidate glioma genes [4]. In order to extend these studies to generate an immunocompetent, autochthonous mouse glioma model useful for glioma gene discovery, we generated mice in which the SB11 version of the transposase is expressed from the human *Glial Fibrillary Acidic Protein* promoter (GFAP-SB11). We found that mobilizing T2 transposons with GFAP-SB11 on an otherwise wild-type background did not promote gliomas, and very rare gliomas were observed when transposons were mobilized on a *p19Arf* ^+/−^ cancer predisposed background. In order to identify additional candidate glioma genes, we studied additional mice undergoing whole-body mutagenesis and identified new gliomas from these mice. Cloning of insertions from these tumors identified additional candidate glioma genes.

## Methods

### Ethics Statement

Mouse work was carried out in strict accordance with the recommendations in the Guide for the Care and Use of Laboratory Animals of the National Institutes of Health and was performed under the review and approval of the University of Wisconsin-Madison Institutional Animal Care And Use Committee. Animal condition was monitored daily by animal care staff and at least four times a week by an author who was blinded to genotype. Mice were euthanized by CO_2_ asphyxiation following AVMA Guidelines for the Euthanasia of Animals when any of the following humane endpoints were met: a body condition score of 2 or below [5], hunching behavior, lethargy, inappetence, failure to groom, progressive ataxia, growth retardation, hydrocephalus, seizure, or paralysis. All efforts were made to minimize suffering.

### Mice

Animals were housed with standard housing and husbandry conditions under specific pathogen free conditions. Animals received standard chow and water *ad libitum*. The only procedures that mice underwent were a tail clipping to provide sufficient genomic DNA for genotyping and an ear notch necessary to distinguish animals from each other. These procedures were carried out prior to weaning. T2/onc2 high-copy (on chromosome 4), T2/onc low copy (lines 68 and 76), Rosa26-SB11, *p19Arf^−/−^*, *Blm^−/−^* and *Csf1^op/op^* (hereafter referred to as *Csf1^−/−^*) mice have been previously described [6]–[10]. In addition, mice harboring a version of T2/onc with translational start sequences engineered into the MSCV LTR (T2/oncATG) [4] were also utilized for some crosses to Rosa26-SB11. To generate mice expressing transposase in the *GFAP* compartment, the pGFAP-SB11 plasmid was constructed by excising *SB11* from pCMV-SB11 by *Sac*II digest and cloning it into pGFAP-Nrf2 [11], [12] that had the *Nrf2* gene removed by *Sac*II digest. The pGFAP-SB11 plasmid DNA was linearized with *Sph*I and *Nde*I, and the 3.9 kb GFAP-SB11 transgene was used for pronuclear injections performed by the UW-Madison Transgenic Animal Facility. Transgenic GFAP-SB11 mice were generated on the FVB/N genetic background. Potential founders were screened by PCR analysis (primers 5′-CAT CGC CAG TCT AGC CCA CT-3′, 5′-ACG TGG TAC TTT CAG GCG TT-3′) flanking the *GFAP* promoter and SB11 gene. Three potential founders were identified, two of which transmitted the GFAP-SB11 transgene to their offspring and were used to establish the transgenic lines utilized for this study.

### Immunohistochemistry and immunofluorescence

At necropsy, brains and other tissues were isolated for analysis. Brains were either fresh frozen in OCT or formalin-fixed, paraffin embedded (FFPE) by the UW Carbone Cancer Center Experimental Pathology facility. For some mice, brains were subdivided into four coronal sections at necropsy, two of which were fresh frozen and two of which were FFPE. Immunohistochemistry for SB11 transposase on FFPE tissues was performed as previously described [3]. Immunofluorescence for SB11 transposase was performed using the same primary and secondary antibodies, and streptavidin FITC (ebiosciences) at 1∶100 dilution was used to detect the biotinylated secondary. Immunofluorescence for GFAP was performed using a polyclonal anti-GFAP antibody (Abcam, ab7260) at 1∶5000 dilution and a Texas Red secondary antibody (Vector Laboratories). For IBA1/FLI1 double staining, FFPE sections were deparaffinized and rehydrated. Antigen retrieval was performed by enzymatic digestion using a 20 µg/ml solution of Proteinase K (from DeadEnd Colorimetric TUNEL kit, Promega) in PBS for 13 minutes at 37°C. Tissue sections were quenched in 3% hydrogen peroxide in methanol for 10 minutes, washed in PBS and then blocked in 10% normal goat serum (NGS) (Vector Labs) containing 0.1% Triton X-100 (Fisher Scientific) for 90 minutes at room temperature. Prior to use, rabbit anti-FLI1 primary antibody (Santa Cruz, sc-356) was diluted 1∶50 in 10% NGS and pre-absorbed on de-paraffinized, rehydrated and Proteinase K treated FFPE tissue sections overnight at 4°C. For the first immunostaining step, following the blocking step, tissues were incubated with the pre-absorbed anti-FLI1 primary antibody overnight at 4°C. This was followed by a 30 minute incubation with a biotin labeled anti-rabbit secondary antibody (Abcam) at 1∶200 dilution at room temperature. The Vectastain ABC reagent (Vector laboratories) was used for antigen signal enhancement and DAB chromogen was used to visualize staining. For the second immunostaining step, the DAB stained tissues were blocked in 10% NGS for 90 minutes at room temperature. This was followed by incubation in rabbit anti-IBA1 (1∶200, WAKO) in 10% NGS overnight at 4°C. Then tissues were treated with biotin labeled anti-rabbit (1∶200, Abcam) secondary antibody at room temperature for 30 minutes. The Vectastain ABC reagent (Vector laboratories) was used for antigen signal enhancement and Vinagreen chromogen (Biocare Medical) was used to visualize staining. Thymus (not shown) was used as a positive control for FLI1 immunostaining. A hematoxylin stain was used to visualize nuclei. For pathological analysis, hematoxylin and eosin stained sections were utilized.

### Excision PCR

PCR was performed as previously described [6] on genomic DNA isolated from brains using the MasterPure Complete DNA and RNA Purification Kit (Epicentre). A control PCR amplifying a portion of the *Rosa26* locus was utilized to control for genomic DNA integrity.

### Endpoint PCR

PCR was performed with primers designed to amplify the insertion site in *Fli1* (5′- GGC TAA GGT GTA TGT AAA CTT CCG-3′ and 5′-TGA TTC AGC CAA ATA ATT CAG GAG G-3′) in the glioma in mouse AR151. PCR was performed utilizing decreasing amounts of input AR151 glioma genomic DNA (1X, 0.2X, 0.1X, 0.05X, 0.025X and 0.0125X). To control for DNA quality, excision PCR was also performed on the same amounts of input genomic DNA.

### Linker-mediated PCR

DNA from tumors identified in FFPE samples was extracted according to the manufacturer's instructions using the QIAampDNA FFPE Tissue Kit (Qiagen) and then amplified using the Illustra GenomiPhi V2 DNA amplification kit (GE Healthcare). For tumors identified in fresh frozen samples, the MasterPure Complete DNA and RNA Purification Kit (Epicentre) was utilized to purify genomic DNA. The restriction digests, linker annealing and primary PCR steps for linker-mediated PCR were performed as previously described [13]. Secondary and tertiary PCRs were used to add barcodes for tumor identification as well as sequences necessary for sequencing on the Illumina platform. Primers for secondary PCR were: Linker nested Illumina primer (5′-CAA GCA GAA GAC GGC ATA CGA GCT CTT CCG ATC TAG GGC TCC GCT TAA GGG AC-3′) and Illumina right T2 barcode primer (5′-CCC TAC ACG ACG CTC TTC CGA TCT X AGG TGT ATG TAA ACT TCC GAC TTC AA-3′) or Illumina left T2 barcode primer (5′- CCC TAC ACG ACG CTC TTC CGA TCT X AAG TGT ATG TAA ACT TCC GAC TTC AA-3′). X indicates the location of a 10–14 base pair barcode unique to each tumor, barcode sequences available upon request. Primers for secondary PCR were: Illumina forward primer (5′-AAT GAT ACG GCG ACC ACC GAG ATC TAC ACT CTT TCC CTA CAC GAC GCT CTT CCG ATC T-3′) and Linker nested Illumina primer. The tertiary PCR reaction was purified for sequencing using the QIAquick PCR purification kit (Qiagen).

### Insertion mapping and CIS analysis

First, sequence reads with valid IRDR sequence were extracted using cross_match. Then, IRDR sequences were removed from these reads and these short reads were aligned against mouse genome assembly (mm9) using GSNAP [14]. Reads reliably aligned to single locus in the genome from GSNAP output were extracted as uniquely aligned reads. Duplicate reads from each library were also removed before CIS analysis. Prior to CIS analysis, insertions sequenced on the Illumina platform were processed as described previously [4] including the removal of insertions residing on the same chromosome as the donor concatemer (“local hops”). Insertions from multiple tumors from the same sequencing method mapping to the same TA were also removed as these are thought to represent PCR artifacts [15]. Filtering of insertions based on read depth (i.e. number of times sequence was read) was performed according to published methods [16], with the exception that there were not a sufficient number of insertions obtained to fit a negative binomial distribution. For all analyses, insertion data was pooled with that from Bender *et al.* [4]. Two approaches were used to analyze insertion data. A previously described method for assigning nonrandom clusters of proviral insertions was used to identify common insertion sites (CISs) using an expected fraction (Efr) of 0.005 and a data set of 2500 insertions. With these criteria, a CIS was defined as 3 insertions from 3 independent tumors within 108 kb or 4 insertions from 4 independent tumors within 351 kb [17]. A gene-centric CIS analysis (gCIS) [16] was also performed. Briefly, the expected number of insertions is based on the number of tumors analyzed, the number of insertion events in UCSC genes in each tumor, and the number of TA di-nucleotides in each gene (i.e. potential insertion sites). A Chi-squared test yields a p-value on the difference of observed transposon insertions per gene versus the expected number. P values are corrected for multiple hypothesis testing using the Bonferroni method. A gene had to be insertionally mutated in 3 or more tumors in order to be a gCIS.

### Comparisons to human datasets

Copy number, DNA methylation, and mRNA expression data from the TCGA's archive of human glioblastomas (GBMs) were examined at those sites prospectively identified as having functional relevance from CIS and gCIS analyses [18]. Frequent mutation, copy number aberrations, or epigenetic silencing events across patient samples were noted as potential evidence for a role in generating cancer phenotypes. TCGA mRNA expression data comparing 542 human GBMs to 10 normal samples at www.oncomine.com was also analyzed for the 20 human orthologs of gCIS/CIS genes represented on the microarray. Data for genes with at least one probe with a Bonferroni corrected p value of <.05 are presented with the gene rank (based on p value) and fold change over normal. When multiple probes for the same gene indicated the same directionality of expression (over-expressed or under-expressed), the greatest fold change is presented. When multiple probes for the same gene indicated different directionalities of expression, “probes discordant” is listed. Ingenuity Pathway Analysis (IPA) (Ingenuity Systems, Inc.) was performed on CIS and gCIS human orthologs. Canonical pathways with Benjamini-Hochberg corrected p values<.05 are reported.

## Results

### Generation of mice expressing transposase in the GFAP compartment

**Figure 1:**
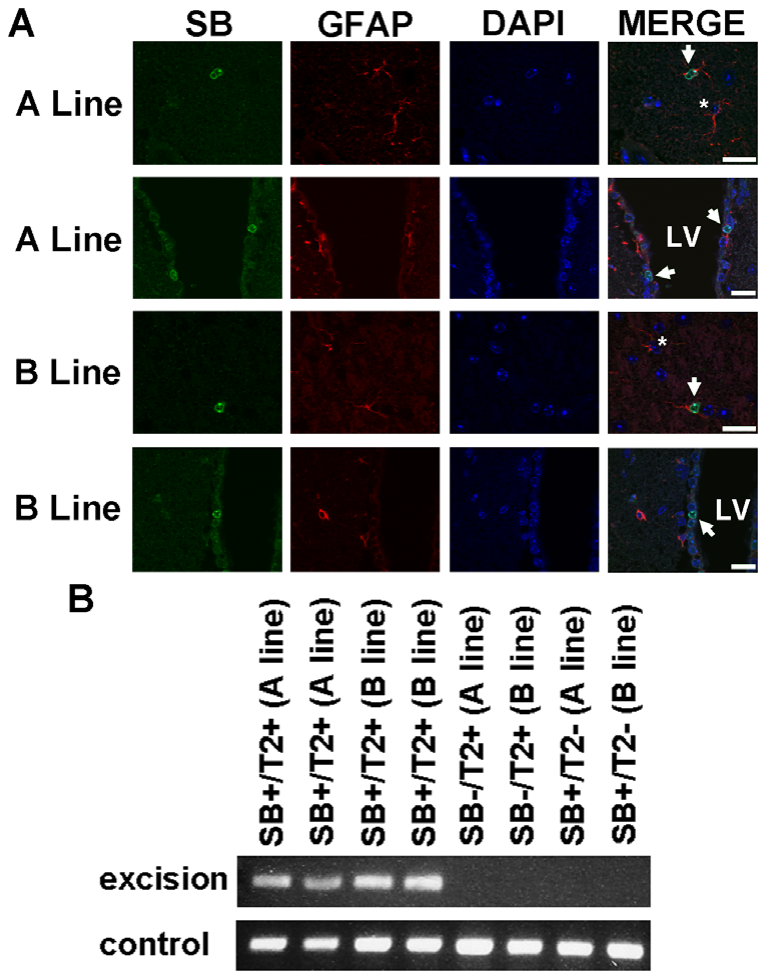
GFAP-SB11 transgenics express functional transposase in a subset of GFAP^+^ cells. Two lines (A and B) were established and used for these experiments. SB = SB transposase, T2 = T2/onc, LV = lateral ventricle. A) Immunofluorescence for GFAP (red) and SB (green). Nuclei are stained with DAPI. Arrows indicate examples of SB^+^ GFAP^+^ cells while asterisks indicate examples of SB^−^ GFAP^+^ cells. Scale bars are 20 µm. B) PCR based excision assay showing that transposons have mobilized in the brains of SB^+^T2^+^ but not SB^−^T2^+^ or SB^+^T2^−^ mice from each line. A control PCR demonstrates that genomic DNA is present for all samples.

To limit transposition to putative glioma-initiating cells, transgenic mice were generated that express the SB11 version of the transposase under the regulation of the human *GFAP* promoter (GFAP-SB11) [19]. The *GFAP* promoter was chosen because GFAP is expressed in both mature astrocytes and neural stem cells (NSCs) in adult mice, both of which have been proposed to be glioma-initiating cells [20], [21]. In addition, GFAP-Cre lines have been used to generate murine glioma models due to conditional deletion of tumor suppressor genes [22]–[24] and astrocytomas form with high penetrance in mice in which activated Ras is expressed from the *GFAP* promoter [25]. Two lines that successfully transmitted the GFAP-SB11 transgene to their progeny were established (A and B). Co-immunofluorescence for transposase and GFAP on brains from adult mice verified that transposase was expressed in a subset of GFAP^+^ cells in both lines (Figure 1A), including a subset of GFAP^+^ cells residing the presumptive stem cell niche in the subventricular zone (SVZ) of the lateral ventricle (LV) (Figure 1A). Immunohistochemistry (IHC) on several tissues revealed that transposase expression was primarily brain specific (Figure S1). To verify that GFAP-SB11 promotes transposition, both GFAP-SB11 lines were crossed to T2/onc transgenics. PCR-based transposon excision assays that detect repaired sites of transposon mobilization from genomic concatemers [6] were used to verify that transposons do mobilize in the brains of GFAP-SB11;T2/onc mice (Figure 1B). Therefore, these results indicate that GFAP-SB11 mice express functional transposase in some, but not all, GFAP^+^ cells. Given their similar transposase expression patterns, data from both lines were combined for analysis below.

### Mobilization of T2/onc by Rosa26-SB11 generates more highly penetrant tumors than does mobilization in the GFAP compartment

To determine if transposon mobilization in the *GFAP* compartment generates sufficient insertional mutagenesis to cause glioma formation, GFAP-SB11 mice were crossed to T2/onc transgenics. For these experiments a high copy (HC) T2/onc2 line harboring >200 copies of T2/onc2 on chromosome 4 [7] was utilized to maximize insertional mutagenesis rates. A cohort of GFAP-SB11; T2/onc2 HC mice were generated and aged. By 18 months, only one of 32 GFAP-SB11; T2/onc2 HC mice had died. The brain from this mouse was not available for analysis. The remaining 31 GFAP-SB11; T2/onc2 HC mice were sacrificed for analysis between 18 and 19 months of age and no gliomas were found. No phenotypes were also observed in control GFAP-SB11 or T2/onc2 HC brains (Table 1). The penetrance of gliomas due to Rosa26-SB11 mobilization of T2/onc was previously found to be greater on a *p19Arf^+/−^* background, so a cohort of *p19Arf^+/^* ^−^; GFAP-SB11; T2/onc mice as well as controls (*p19Arf^+/−^*; GFAP-SB11 and *p19Arf^+/−^*; T2/onc) were generated. For this study, some mice harbored the HC T2/onc2 line and some harbored a low-copy T2/onc line (LC76). Data from both transposon lines were combined for analysis and hereafter will be referred to as T2/onc for simplicity. By one year of age, a limited number of *p19Arf^+/−^*; GFAP-SB11; T2/onc mice had died or become moribund, however there was no statistically significant difference in survival compared to *p19Arf^+/−^* mice without mobilizing transposons (Figure S2). *p19Arf^+/−^* mice with and without mobilizing transposons were pathologically examined for tumors. Two gliomas were found in *p19Arf^+/−^*; GFAP-SB11; T2/onc mice (n = 80), but none were found in control *p19Arf^+/−^* mice (n = 79) (Table 1, Figure S3). One of the gliomas was observed in a mouse harboring T2/onc LC, while one was observed in a mouse harboring T2/onc2 HC. DNA of sufficient quality for cloning insertions was obtained from one glioma (see below). Hypercellularity was observed in the brains of five mice, however these included both mice with and without mobilizing transposons.

**Table 1:** Genotypes and phenotypes of analyzed brains from GFAP-SB11 crosses.

GFAP-SB^a^	T2	p19	# analyzed	# of gliomas	# of other phenotypes
+	+	wt	31	0	1 lipoma
+	−	wt	9	0	0
−	+	wt	6	0	0
+	+	+/−	80	2 (1 low grade, 1 grade undetermined)	2 hypercellular, 1 brain calcifications, 1 hematopoietic cell infiltrate
+	−	+/−	21	0	1 hypercellular, 1 hematopoietic cell infiltrate
−	+	+/−	58	0	2 hypercellular

a Abbreviations used: GFAP-SB = GFAP-SB11 transposase, T2 = T2/onc, p19 = p19Arf, wt = wild-type.

In order to identify additional SB-induced or accelerated gliomas for study, we pathologically analyzed brains isolated from additional moribund mice in which transposons were mobilized from low copy (LC) T2/onc lines throughout the body by Rosa26-SB11 (Table 2). Mice presented in Table 2 have not been previously analyzed and therefore are unique to this study. T2/onc LC lines were utilized for these studies as mobilization of T2/onc2 from HC lines by Rosa26-SB11 results in high levels of embryonic lethality [7]. Mice for our study included Rosa26-SB11; T2/onc mice on an otherwise wild-type genetic background as well as on the *p19Arf^+/−^*, *p19Arf^−/−^*, *Blm^+/−^* and *Blm^−/−^* genetic backgrounds. In addition, we analyzed mice in which a T2/onc transposon containing translational start sequences in the MSCV LTR over-expression element was mobilized by Rosa26-SB11 [4]. As *Csf1* was the gene insertionally mutated the most frequently in our previous SB glioma screen [4], we hypothesized that identification of additional glioma genes would be facilitated on a *Csf1* deficient or heterozygous background. Unfortunately, both *Csf1^−/−^*; Rosa26-SB11; T2/onc and *Csf1^+/−^*; Rosa26-SB11; T2/onc mice were born at less than Mendelian ratios (p<.0001, Chi square analysis). Therefore, only a limited number of brains from *Csf1^−/−^* or *Csf1^+/−^* mice with mobilizing transposons were available for analysis. Overall, gliomas were observed at low penetrance (15 out of 199 total mice with transposons mobilized by Rosa26-SB11; Table 2 and Figure S3). The presence of infiltrating hematopoietic cells on the brain surface was also observed in some mice (Table 2), which is not unexpected given that SB-driven leukemia/lymphoma is commonly observed when T2/onc transposons are mobilized by Rosa26-SB11 and is frequently the cause of morbidity in these mice [3]. With the exception of the *p19Arf^−/−^* deficient background, gliomas were not observed in control mice without mobilizing transposons that were studied (Table 2 and [4]). Genomic DNA of sufficient quality for cloning insertions was obtained from 11 gliomas with mobilizing transposons.

**Table 2:** Genotypes and phenotypes of analyzed brains from Rosa26-SB11 crosses.

Rosa-SB11	T2LC^a^	T2ATG	p19	Blm	Csf	# analyzed	# of gliomas	# of other phenotypes
Mice with mobilizing transposons:								
+	+	−	wt	wt	wt	49	3 (1AA, 2 grade undetermined)	6 hematopoietic cell infiltrate, 1 hypercellular
+	−	+	wt	wt	wt	66	3 (1 AA, 1 low grade, 1 grade undetermined)	11 hematopoietic cell infiltrate, 5 hypercellular,1 calcifications
+	+	−	+/−	wt	wt	9	1 low grade	1 hematopoietic cell infiltrate
+	−	+	+/−	wt	wt	25	1 AA	7 hematopoietic cell infiltrate
+	+	−	−/−	wt	wt	3	1 (grade undetermined)	1 hematopoietic cell infiltrate or DD PNET
+	+	−	wt	+/−	wt	5	1 (AA)	1 hematopoietic cell infiltrate
+	+	−	wt	−/−	wt	13	3 (2 AA, 1 grade undetermined)	4 hematopoietic cell infiltrate, 1 hypercellular with calcifications
+	+	−	wt	wt	+/−	23	2 (1 grade undetermined, 1 GBM)	2 hematopoietic cell infiltrate, 2 hypercellular, 1 inflammatory focus, 1 ischemia
+	+	−	wt	wt	−/−	6	none	1 ischemia
Control mice without mobilizing transposons:								
−	+	−	wt	wt	wt	7	none	none
+	−	−	wt	wt	wt	7	none	none
−	−	−	wt	wt	wt	2	none	none
−	+	−	−/−	wt	wt	6	none	1 pituitary tumor DD tumor with neuronal differentiation DD metastasis of other primary tumor
+	−	−	−/−	wt	wt	11	2 (GBM)	1 possible PNET
−	−	−	−/−	wt	wt	6	none	1 hematopoietic cell infiltrate
−	+	−	wt	−/−	wt	1	none	none
+	−	−	wt	−/−	wt	2	none	none
−	−	−	wt	−/−	wt	2	none	none
−	+	−	wt	wt	+/−	1	none	none
+	−	−	wt	wt	+/−	1	none	none
−	+	−	wt	wt	−/−	1	none	none
+	−	−	wt	wt	−/−	1	none	none

a Abbreviations used: T2LC = T2/onc LC, T2ATG = T2/oncATG, p19 = p19Arf, wt = wild-type, AA = anaplastic astrocytoma, GBM = glioblastoma, PNET = primitive neuroectodermal tumor, DD = differential diagnosis.

### CIS analysis identifies candidate glioma genes

For high-throughput transposon insertion site cloning from gliomas, linker-mediated PCR protocols were modified to be compatible with sequencing on the Illumina platform. In addition to the one GFAP-SB11 tumor and the 11 new Rosa26-SB11 tumors described above, insertions were re-cloned from DNA isolated from frozen sections of two tumors (68R544 and 76R339p19) previously studied utilizing DNA isolated from paraffin sections [4], bringing the total number of tumors analyzed on the Illumina platform to 14 (Table S1). After processing of insertions as described in the Methods, 1385 insertions were identified. To increase our ability to identify CISs [26], insertion data from the new gliomas identified above were combined with insertion data from our previous study on SB gliomas [4] yielding a total dataset of 2257 unique insertions (Table S2) from 33 gliomas. This combined dataset was used for all further analyses.

In our previous glioma study, CISs were defined based on insertion site clustering using criteria previously applied to retroviral mutagenesis. Applying the same criteria to the dataset of 2257 insertions and requiring at least three individual tumors to define a CIS identified 28 CISs. Two (*Sfi1* and *No gene chromosome 2* (*NG2*)) were found in an unselected SB insertion dataset [3] and therefore were removed from further consideration, bringing the total to 26. These included three of four CISs identified in our previous study (*Csf1*, *Fli1*, and *Mkln1*) as well as 23 additional CISs (Table 3). Insertions in *Csf1*, *Fli1* and *Mkln1* were present in tumors previously studied in Bender *et al.* as well as novel tumors isolated in the current study. As this method of CIS identification detects a high level of false positives [17], more stringent gene-centric (gCIS analysis) [16] was performed. Seven gCISs were identified. *Sfi1* was also identified by this method and as it was also found to be a gCIS in a non-tumorigenic brain SB insertion dataset [27], was removed from further consideration. The remaining six gCISs (Table 3) over-lapped with CISs identified by insertion clustering. Three gCISs (*Csf1*, *Fli1* and *Mkln1*) had been identified as CISs in our previous study. Novel gCIS are *GM1647*, *Ppp3r1* and *Elovl6*. Additional details about CISs/gCISs are presented in Table S3.

**Table 3:** CISs and gCISs identified in SB-gliomas.

Gene	GB^a^ accession #	Method	p value	# of tumors	Additional genes in CIS
Nav3	NM_001081035	CIS	N/A	3	NONE
Ppp3r1	NM_024459	gCIS, CIS	2.16E-08	3(gCIS), 5(CIS)	Plek, Cnrip1, Pno1, Wdr92, C1d
Tanc2	NM_181071	CIS	N/A	3	NONE
Rps6ka5	NM_153587	CIS	N/A	4	NONE
Crebbp	NM_001025432	CIS	N/A	3	NONE
Lsamp	NM_175548	CIS	N/A	4	NONE
Gramd1c	NM_001172107	CIS	N/A	4	2610015P09Rik, Zdhhc23, AK016097, Atp6v1a, BC050135, Naa50
Dyrk1a	NM_007890	CIS	N/A	4	NONE
Arid1b	NM_001085355	CIS	N/A	4	NONE
Smad4	NM_008540	CIS	N/A	3	NONE
Abtb2	NM_178890	CIS	N/A	4	Caprin 1, Nat10, 4930547E08Rik
Pag1	NM_001195031	CIS	N/A	3	NONE
Kpna4 (CIS)/Gm1647(gCIS)	NM_008467/NM_001243000	gCIS, CIS	4.40E-13	3	NONE
Csf1	NM_007778	gCIS, CIS	0	17	Slc6a17,Ubl4b, Alx3, Strip1, Ahcyl1
Elovl6	NM_130450	gCIS, CIS	2.39E-05	3	NONE
Chd7	NM_001277149	CIS	N/A	4	NONE
Nfia	NM_001122952	CIS	N/A	3	NONE
Faf1	NM_007983	CIS	N/A	3	NONE
Rbpj	NM_001080928	CIS	N/A	4	Cckar, AK164362, Tbc1d19
Hepacam2	NM_178899	CIS	N/A	3	Ccdc132
Mkln1	NM_013791	gCIS, CIS	1.63E-06	6(CIS), 4(gCIS)	Lincpint, 2210408F21Rik, Mkln1os
Zfp212	NM_145576	CIS	N/A	4	Pdia4, Zfp786, Zfp398, Zfp282, Zfp783, Zfp956, Zfp777, Zfp746
Tsg101	NM_021884	CIS	N/A	3	Ldha, Ldhc, Uevld
Mir101c	NR_039546	CIS	N/A	3	NONE
Fli1	NM_008026	gCIS, CIS	3.63E-20	6	NONE
Usp9x	NM_009481	CIS	N/A	3	NONE

a Abbreviations used: GB = GenBank, N/A = not applicable, p value = p value for gCIS.

To determine if CISs and gCISs identify candidate processes (gene networks and signaling pathways) involved in gliomagenesis, IPA analysis was performed. “Connective Tissue Development and Function, Embryonic Development, Organ Development”, “Behavior, Cellular Development, Cellular Growth and Proliferation”, and “Cell Cycle, Hematological System Development and Function, Humoral Immune Response” were the three gene networks enriched in the CIS gene list. “Lipid metabolism, small molecule biochemistry, inflammatory disease” was the gene network enriched in the gCIS list, with all 5 gCISs with human orthologs being found in this network. The signaling pathway enriched in the CIS gene list was “Glucocorticoid receptor signaling” (p value.039). “Role of osteoblasts, osteoclasts, and chondrocytes in rheumatoid arthritis” (p value.0427), “Role of macrophages, fibroblasts, and endothelial cells in rheumatoid arthritis” (p value.0427) and “Hematopoiesis from multipotent stem cells” (p value.0427) were the signaling pathways enriched for gCIS genes. These analyses support the hypothesis that SB mutagenesis in an autochthonous immunocompetent model identifies processes involved in glioma cells themselves and in modulating the activity of cells in the tumor microenvironment such as immune cells. Given that 32 of 33 SB-gliomas studied are from mice undergoing whole-body mutagenesis it is possible that some CISs/gCISs are due to mutagenesis in the tumor microenvironment.

### FLI1 is expressed in a subset of cells in a glioma harboring an insertion in *Fli1*

**Figure 2:**
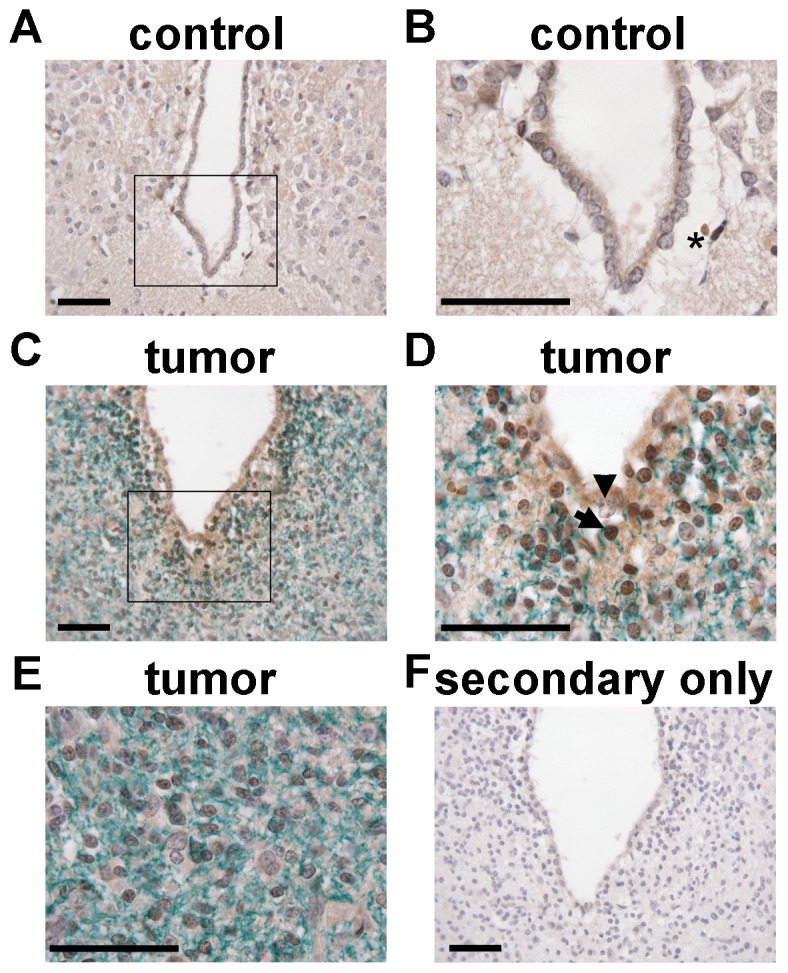
FLI1 is expressed in a subset of cells in a glioma with a *Fli1* insertion. Immunoreactivity for FLI1 is in brown and immunoreactivity for IBA1 is in green. Nuclei are counterstained blue. A) 40× image of the dorsal third ventricular region in a control mouse without mobilizing transposons. B) 100× image of the boxed area in A. Asterisk indicates a FLI1 immunoreactive cell with morphologic features of a red blood cell. C) 40× image of the dorsal third ventricular region surrounded by tumor in AR151. D) 100× image of the boxed area in C. Arrowhead points to a nucleus that is negative for FLI1 and an arrow indicates an example of strong nuclear FLI1 staining. E) 100× image of tumor in AR151 that is distant from the ventricle. F) A 40× image of secondary only controls is shown for comparison to verify specific primary antibody staining. Scale bars = 50 µm.

The gCIS genes *Csf1* and *Fli1* have been implicated in controlling macrophage numbers [28], [29]. For *Fli1*, all transposon insertions in gliomas are clustered in intron 1 or 2 in the same orientation of the gene (Table S2) and therefore are predicted to cause over-expression of a N-terminally truncated protein that contains the pointed and ETS domains. To further explore the involvement of *Fli1* in SB-induced gliomas, we performed dual IHC for FLI1 and IBA1 (a marker for macrophages including brain resident microglia) on AR151, the glioma harboring an insertion in *Fli1* for which FFPE material suitable for IHC was available. Insertions in both *Csf1* and *Fli1* were cloned from sections of fresh frozen tumor found in this mouse (see methods). In addition, this mouse did not have enlarged hematopoietic organs at necropsy and pathologic examination did not find any evidence of hematologic disease, indicating that these insertions do not likely result from CNS infiltration of leukemia cells. In the AR151 glioma, strong nuclear FLI1 immunoreactivity was detected in a subset of cells in the tumor adjacent to the dorsal third ventricle (brown staining, Figure 2C and D). FLI1 immunoreactivity in the same region of non-tumor bearing mice (brown staining, Figure 2A and B) primarily occurred in cells with morphologic characteristics of red blood cells. Significantly greater immunoreactivity for IBA1 was also detected in the glioma (green staining, Figure 2C and D) compared to control brain (green staining, Figure 2A and B), indicating tumor infiltration by microglia and/or peripherally derived macrophages. However, the nuclear localization of FLI1 and the cytoplasmic localization of IBA1 did not allow us to determine if their expression co-localized. Therefore, although FLI1 expression can be detected in a tumor with an insertion in *Fli1*, expression is limited to a subset of cells in a specific region of the tumor. Furthermore, “endpoint” PCR [30] to detect the transposon insertion in *Fli1* in this tumor only generated a product with the highest amounts of input tumor genomic DNA tested (Figure S4), also indicating that the insertion is not highly clonal within the tumor.

### Comparison with human tumor genetic, epigenetic and expression data implicate a subset of gCISs and CISs as drivers in human GBM

To investigate if gCISs and CISs identify genes that are genetically or epigenetically modified during human GBM development, TCGA GBM exome re-sequencing (n = 291 tumors), methylation (n = 353) and copy number (n = 542) data were analyzed (Table 4). In addition, the TCGA mRNA microarray expression dataset comparing GBM to normal brain was also examined to determine if gCISs and CISs commonly have altered expression in human GBM. The only non-synonymous point mutation in a gCISs in human GBM was found in *FLI1*. However, *FLI1* was hypomethylated in over 60% of GBMs and microarray data indicate that it is over-expressed on the mRNA level in GBM compared to normal brain. Previous qRT-PCR and immunohistochemical analyses have detected increased levels of CSF1 in human high-grade gliomas [4], [31]. As *CSF1* was hypomethylated in over 90% of GBMs and had copy number gain in a subset of GBMs, both epigenetic and genetic mechanisms may contribute to the increased expression of *CSF1* observed in human tumors. The gCIS gene *MKLN1* is located on chromosome 7, and frequently has copy number gain in GBM. In addition, *MKLN1* is hypomethylated in human GBM and it is in the top 5% of over-expressed genes in the human GBM microarray dataset. Therefore, *MKLN1* is a candidate driver of human glioma development. The other gCISs, *ELOVL6* and *PPP3R1* are frequently hypomethylated in GBM. Microarray data indicated that *ELOVL6* is over-expressed in human GBM compared to normal brain, however it was only in the top 29% of genes based on p value. Microarray data indicate that *PPP3R1* is under-expressed in human GBM compared to normal brain.

**Table 4:** The mutational, copy number, methylation and mRNA expression status of gCIS/CIS human orthologs in TCGA GBM data.

Gene	% point mutations	% deleted/lost	% amplified/gained	% Hyper-methylated^c^	% Hypo-methylated	mRNA
Nav3	1.03%	8.12%	7.01%	7.93%	0.00%	top 1%, −11.076 fc
Ppp3r1	0.00%	4.61%	3.32%	18.98%	79.04%	top 8%, −4.583 fc
Tanc2	1.37%	3.51%	6.83%	0.85%	0.00%	N/A
Rps6ka5	0.34%	24.91%	2.58%	0.00%	99.43%	top 7%, −3.370 fc
Crebbp	1.72%	5.90%	4.24%	59.21% (2.55%)	18.13%	probes discordant
Lsamp	0.00%	9.96%	7.01%	2.27% (1.42%)	82.44%	top 5%, −2.370 fc
Gramd1c	0.00%	7.56%	7.56%	20.40%	0.00%	ns
Dyrk1a	0.00%	5.35%	7.01%	20.11%	78.75%	top 21%, −1.405 fc
Arid1b	0.34%	23.25%	2.58%	N/A	N/A	N/A
Smad4	0.34%	9.04%	6.46%	0.00%	99.72%	top 3%, 3.271 fc
Abtb2	0.00%	13.10%	2.58%	13.03%	75.64%	top 27%, −1.434 fc
Pag1	0.00%	4.24%	8.49%	N/A	N/A	N/A
Kpna4 (CIS)/Gm1647(gCIS)^a^	0.00%	6.09%	8.49%	0.00%	20.68%	top 25%, 1.603 fc
Csf1	0.00%	2.77%	11.25%	0.00%	99.15%	top 24%, 1.380 fc
Elovl6	0.00%	4.80%	2.77%	0.00%	99.72%	top 29%, 1.710 fc
Chd7	0.69%	4.98%	7.56%	3.40%	0.00%	top 20%, 2.243 fc
Nfia	0.34%	2.21%	12.36%	N/A	N/A	N/A
Faf1	0.34%	10.15%	9.78%	1.70%	67.42%	ns
Rbpj	0.00%	5.72%	3.51%	5.95%	79.60%	top 16%, 1.984 fc
Hepacam2	0.69%	N/A	N/A	N/A	N/A	N/A
Mkln1	0.00%	0.55%	78.41%	0.00%	79.04%	top 5%, 1.898 fc
Zfp212	0.00%	1.11%	76.57%	0.00%	99.72%	ns
Tsg101	0.00%	13.47%	1.85%	0.00%	99.72%	ns
Mir101c	N/A^b^	N/A	N/A	N/A	N/A	N/A
Fli1	0.34%	11.81%	2.77%	20.40%	63.46%	top 11%, 2.523 fc
Usp9x	0.69%	0.92%	37.64%	69.12%	20.68%	top 25%, −1.202 fc

a For *Kpna4/Gm1647* data presented are for *KPNA4* as *Gm1647* does not have a human ortholog.

b Abbreviations used: N/A = not applicable (no human ortholog, or data not available), ns = non-significant, fc = fold change.

c The percentage of tumors harboring hypermethylation and evidence for decreased mRNA expression (epigenetic silencing) are shown in parentheses.

Of the CIS genes, *CREBBP* was the most commonly mutated in GBM, with 1.72% of tumors harboring non-synonymous mutations. Deletions in *ARID1B* are observed in GBM (Table 4 and [32]), indicating it is a candidate tumor suppressor gene. Although deletions involving *CREBBP* were only observed in approximately 6% of GBM, the *CREBBP* locus was frequently hypermethylated in human GBMs, and for 9 tumors this was correlated with a significant down-regulation of mRNA expression (i.e. epigenetic silencing). Therefore, *CREBBP* is implicated as a novel candidate tumor suppressor gene in a subset of GBMs. The CIS *RPS6KA5* was deleted in approximately 25% of GBMs and was also found in the top 7% of genes down-regulated at the mRNA level in the microarray dataset, implicating it as a candidate tumor suppressor gene. In addition, genes with documented roles in gliomagenesis such as *FAF1* [33], *DYRK1A* [34] and *SMAD4* [35] were identified as CISs in this screen.

## Discussion

In these studies, transposon mobilization in the *GFAP* compartment failed to generate gliomas with substantial penetrance, even in a *p19Arf* heterozygous background. There are several possible explanations for this result. First, although transposase expression was detected in a subset of GFAP^+^ cells in these mice, it is possible that the rate of mutagenesis or the number of cells undergoing mutagenesis was too low to cause overt tumor formation. It is also possible that gliomas are observed when Rosa26-SB11 promotes mobilization because mutagenesis in the stroma also promotes glioma development. In support of the former possibility, it was reported that NSCs isolated from mice in which SB11 was mobilized in the *Nestin* compartment were capable of immortalization with an astroglial like phenotype *in vitro* and generated tumors when injected into the flanks of immunocompromised mice. However, significant rates of insertional mutagenesis were likely required as NSCs were cultured for 2–3 months before they became immortalized and immortalized cells took another 2 months to establish gliomas in immunocompromised hosts [27].

With the exception of the *p19Arf^−/−^* background, no gliomas were observed in controls that lacked mobilizing transposons (Table 1,2). Although deletions at the *CDKN2A* locus that includes *ARF* and *INK4A* commonly occur in human high-grade gliomas, gliomas have not been previously reported in mice deficient for *p19Arf* [8]. Two glioblastomas were observed in control *p19Arf^−/−^* mice in our studies. It is possible that genetic modifiers present on the C57Bl/6 and FVB/N mixed strain background generated during the breedings for our studies can cooperate with *p19Arf* deficiency to cause gliomagenesis. Alternatively, because both mice with gliomas were positive for Rosa26-SB11, it cannot be definitively ruled out that transposase expression does not contribute to the observed phenotype.

Although autochthonous, immunocompetent models best model processes that occur during tumorigenesis in humans, the diffuse and infiltrative nature of gliomas in our studies made them difficult to identify at necropsy. Therefore, tumors were identified from histologic slides and genomic DNA for insertion site cloning was isolated from adjacent sections. Some gliomas were identified from FFPE tissue samples while others were identified in samples fresh frozen in OCT. For FFPE samples, DNA quality limited the number of insertions cloned from these tumors, even with whole genome amplification (Table S1). Although higher-quality DNA was obtained from gliomas from fresh frozen samples, insertion read depth was not as great as those from solid tumors or hematopoietic lymphomas that are easily identifiable at necropsy. This can impact gCIS analysis, as low read depth decreases accuracy and especially sensitivity of gCIS detection [16]. Both the penetrance of gliomas and the limited amount of genomic DNA that can be isolated from identified tumors limit the utility of this approach for high-throughput studies.

There was no overlap of gCISs and minimal overlap of CISs compared to gCISs obtained from immortalized astroglial-like cells or their derivative gliomas generated from mice undergoing SB mutagenesis in the *Nestin* compartment (Table S3) [27]. There are several possibilities to explain this difference. All but one glioma utilized for insertion site cloning in our study were from Rosa26-SB11 mice in which transposase is expressed in most cells of the body, therefore it is possible that gliomas in Rosa26-SB11 mice arise from a different cell type than gliomas derived from Nestin^+^ cells. It is also possible that stromal mutagenesis contributed to tumorigenesis in our model. In addition, as mutagenesis in our model was performed *in vivo* in an immunocompetent setting it is also possible that genes identified are involved in modulating tumor/immune interactions. In support of this, *Csf1* was the gene most frequently insertionally mutated in Rosa26-SB11 gliomas in both this and our previous study. In this study, the glioma from a *Csf1* heterozygous mouse also harbored an insertion in *Csf1*, indicating a strong selective pressure for *Csf1* up-regulation during Rosa26-SB11-driven gliomagenesis. We, and others, have demonstrated high levels of *CSF1* mRNA and protein in human gliomas [4], [31] and we present here that both genetic and epigenetic changes occur at the *CSF1* locus in human GBM.

The second most commonly insertionally mutated gene in Rosa26-SB11 gliomas was *Fli1* (6 total tumors). *Fli1* is highly expressed in endothelial and hematopoietic cells and was originally identified as a common integration site in retroviral-induced murine erythroleukemias [36]. Moreover, it is also a CIS in Rosa26-SB11-driven lymphocytic leukemias [3]. As many Rosa26-SB11; T2onc mice with gliomas also have leukemias, it is possible that *Fli1* insertions cloned from gliomas actually come from CNS infiltrating leukemia cells. However AR151 did not have leukemia and did have a *Fli1* glioma insertion. Loss- and gain-of-function genetic studies have implicated *FLI1* in regulating the numbers and/or activity of both lymphoid and myeloid lineage cells. For example, FLI1 is expressed in cultured monocytes and macrophages, and modulates their response to an inflammatory stimulus [37], [38]. It has recently been shown that a subset of macrophages recruited to spinal cord injury migrate into the CNS through the choroid plexuses and the cerebrospinal fluid [39]. Therefore, FLI1^+^ cells observed adjacent to the ventricle could represent macrophages or other immune cells invading from the periphery into the tumor. In the TCGA microarray study, *FLI1* was found to be over-expressed on the mRNA level (Table 4). However, a previous immunohistochemical study of human GBM found FLI1 protein expression in 1 of 40 tumors [40]. It is possible that FLI1^+^ cells in human gliomas would be regionally restricted and present in tumor biopsies utilized for genomics/expression studies, but not always detected in the small cores present in tumor microarrays utilized for immunohistochemical studies.

MKLN1 (muskelin1) is highly expressed in the hippocampus and cerebellum [41] and has known roles in intracellular transport and regulation of cell spreading [42], [43]. In humans, *MKLN1* is located on chromosome 7, and frequently has copy number gain in GBM [44] (Table 4). In addition, the *MKLN1* locus is frequently hypomethylated and it is over-expressed at the mRNA level in GBMs compared to normal brain (Table 4), therefore implicating *MKLN1* as a glioma oncogene. The B1 regulatory subunit of calcineurin, *Ppp3r1*, was a gCIS in our screen and the catalytic subunit *Ppp3ca* was insertionally mutated in two tumors as well (Table S2). Recently, the catalytic subunit of calcineurin was shown by IHC to be expressed in regions of high infiltration/migration in human GBMs [45], however microarray data indicate that *PPP3R1* (Table 4) and *PPP3CA* (not shown) are under-expressed in human GBM compared to normal brain. Therefore further experiments will be necessary to characterize the contribution of calcineurin to gliomagenesis. *Elovl6* (elongation of long-chain fatty acids family member 6) is highly expressed in the brain [46]. It catalyzes the elongation reaction of palmitate to stearate and therefore its expression levels impact fatty acid composition [47]. It is over-expressed in pediatric germinomas [48] and microarray data indicate increased expression in GBM as well. Our analysis implicated *CREBBP*, a histone acetyltransferase involved in chromatin remodeling, as a novel tumor suppressor in human glioma. Deletions and/or mutations in *CREBBP* occur in several cancer types including certain leukemia and lymphoma subtypes, adenoid cystic carcinoma, transitional cell carcinoma, small-cell lung cancer, esophageal squamous cell carcinoma and medulloblastoma [49]–[63]. A low percentage of GBMs harbor point mutations in *CREBBP*, however there is evidence for epigenetic regulation of *CREBBP* in GBM as well as deletion at the *CREBBP* locus in a small percentage of tumors. Our data indicate that additional studies of the role of *CREBBP* in gliomagenesis are warranted.

In summary, transposon mobilization in the *GFAP* compartment was insufficient to drive glioma formation. However, the identification of additional gliomas in mice undergoing whole-body transposon mutagenesis allowed us to identify candidate glioma genes, and analysis of human glioma genetic, methylation and mRNA expression data implicate several of these genes as functioning as drivers during gliomagenesis. Future work will be required to determine the contributions of these genes to gliomagenesis.

## Supporting Information

Figure S1 **GFAP-SB11 expression is tissue specific.** Immunohistochemistry for SB transposase (brown) for both transgenic lines utilized (A and B) shows specific nuclear staining in the brain but not other tissues. Secondary only controls are shown for comparison to define non-specific staining. Scale bar = 100 µm.
(TIF)

Figure S2 **Transposon mobilization by GFAP-SB11 does not accelerate time to morbidity in** ***p19Arf^+/−^*** **mice.** Kaplan Meier survival curve showing time to morbidity of *p19Arf^+/−^* mice with GFAP-SB11 mobilizing transposons (p19^+/−^; T2^+^; SB^+^, squares) is not statistically different (p = .1772, Logrank test) than that of control *p19Arf^+/−^* mice with GFAP-SB11 only (p19^+/−^; T2^−^; SB^+^, circles) or transposons only (p19^+/−^; T2^+^; SB^−^, triangles). Data from GFAP-SB11 A and B lines as well as two different transposon lines (T2/onc LC76 and T2/onc2 HC) were combined for analysis.
(TIF)

Figure S3 **Examples of brain phenotypes from mice with mobilizing transposons.** A) Hematoxylin and eosin (H&E) stained section of a low-grade glioma from a *p19Arf^+/−^*; GFAP-SB11; T2/onc mouse. B) H&E stained section of a low-grade glioma, characterized by low cellularity and no obvious mitotic activity, from a Rosa26-SB11; T2/onc mouse. Asterisks indicate ill-defined border of normal brain with tumor in each panel. Scale bar = 100 µm.
(TIF)

Figure S4 **Endpoint PCR for the transposon insertion in** ***Fli1*** **in the glioma in AR151.** Decreasing amounts of AR151 glioma genomic DNA were used as input for the PCR. Excision PCR was used to control for genomic DNA quality, while genomic DNA from a T2/onc^−^; RosaSB11^−^ (T2^−^SB^−^) mouse and water only controlled for PCR specificity. The *Fli1* insertion could only be detected with high levels of input glioma genomic DNA, indicating that it is present in only a subset of cells within the tumor.
(TIF)

Table S1 **Genotypes of mice from which insertions were cloned from gliomas.** The glioma grade, method of tissue preservation (frozen/FFPE) and number of non-local insertions cloned are also presented. FFPE = formalin fixed paraffin embedded. Tumors that were previously studied using paraffin sections in Bender *et al.* [4] and were re-analyzed using frozen sections are indicated with grey shading. All other tumors are unique to the current study.
(XLSX)

Table S2 **Insertions cloned from SB gliomas.** Insertions from Bender *et al.* [4] are shaded light grey, with the exception of insertions overlapping with those from re-sequencing on the Illumina platform which are underlined. The closest gene within 100 kb is reported. Orientation refers to the direction of transcription of the closest given relative to the promoter in T2/onc. 454 = 454 sequencing platform, SG = shot gun (Sanger sequencing of TA-cloned products), IL = Illumina, Chr = chromosome, N/A = not applicable, CDS = coding sequence.
(XLSX)

Table S3 **CIS/gCIS GenBank accession numbers, genomic locations, and comparisons to previous SB studies.** The GenBank accession numbers (#) for gCIS/CIS genes and the mouse chromosomal (chr) locations of gCIS/CIS insertions are shown. Comparisons to gCISs/CISs identified in immortalized astroglial-like cells (IMM) and their derivative tumors (TUMOR) from Koso *et al* [27] and gliomas from our previous study (Bender *et al.*) [4] are also shown.
(XLSX)
