# Supplementary figures and images for: Sleeping Beauty Mouse Models Identify Candidate Genes Involved in Gliomagenesis
Source: PLoS One. 2014 Nov 25;9(11):e113489. doi: 10.1371/journal.pone.0113489 (PMC4244117; doi:10.1371/journal.pone.0113489)

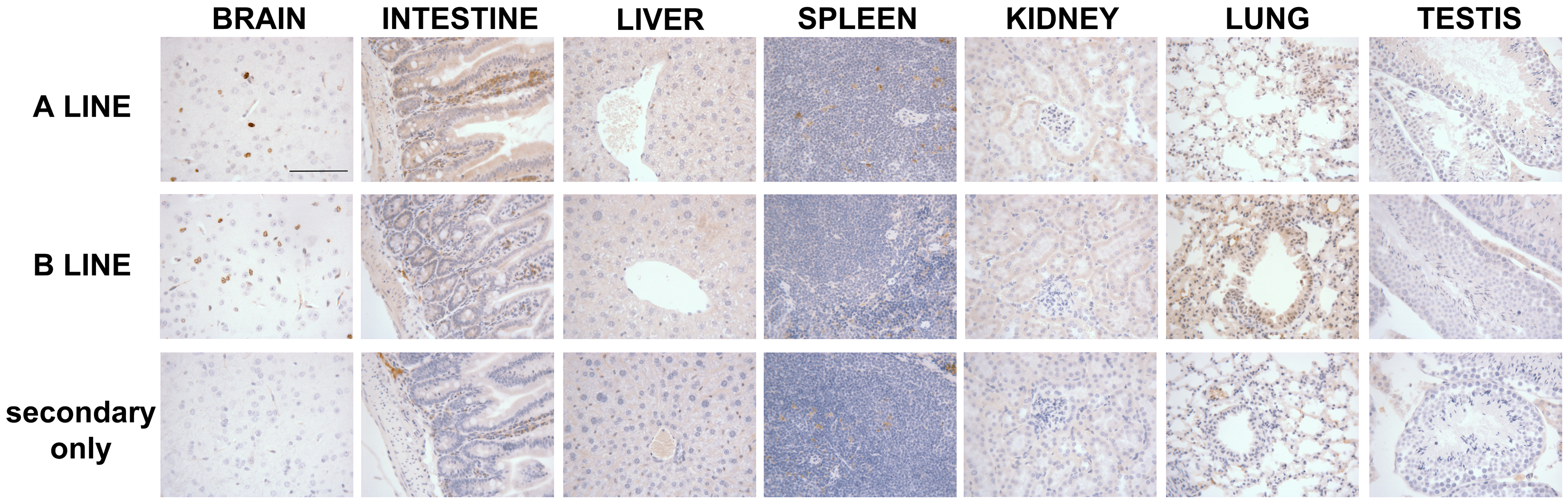

Supplement: Figure S1 — GFAP-SB11 expression is tissue specific. Immunohistochemistry for SB transposase (brown) for both transgenic lines utilized (A and B) shows specific nuclear staining in the brain but not other tissues. Secondary only controls are shown for comparison to define non-specific staining. Scale bar = 100 µm. (TIF) [file pone.0113489.s001.tif]

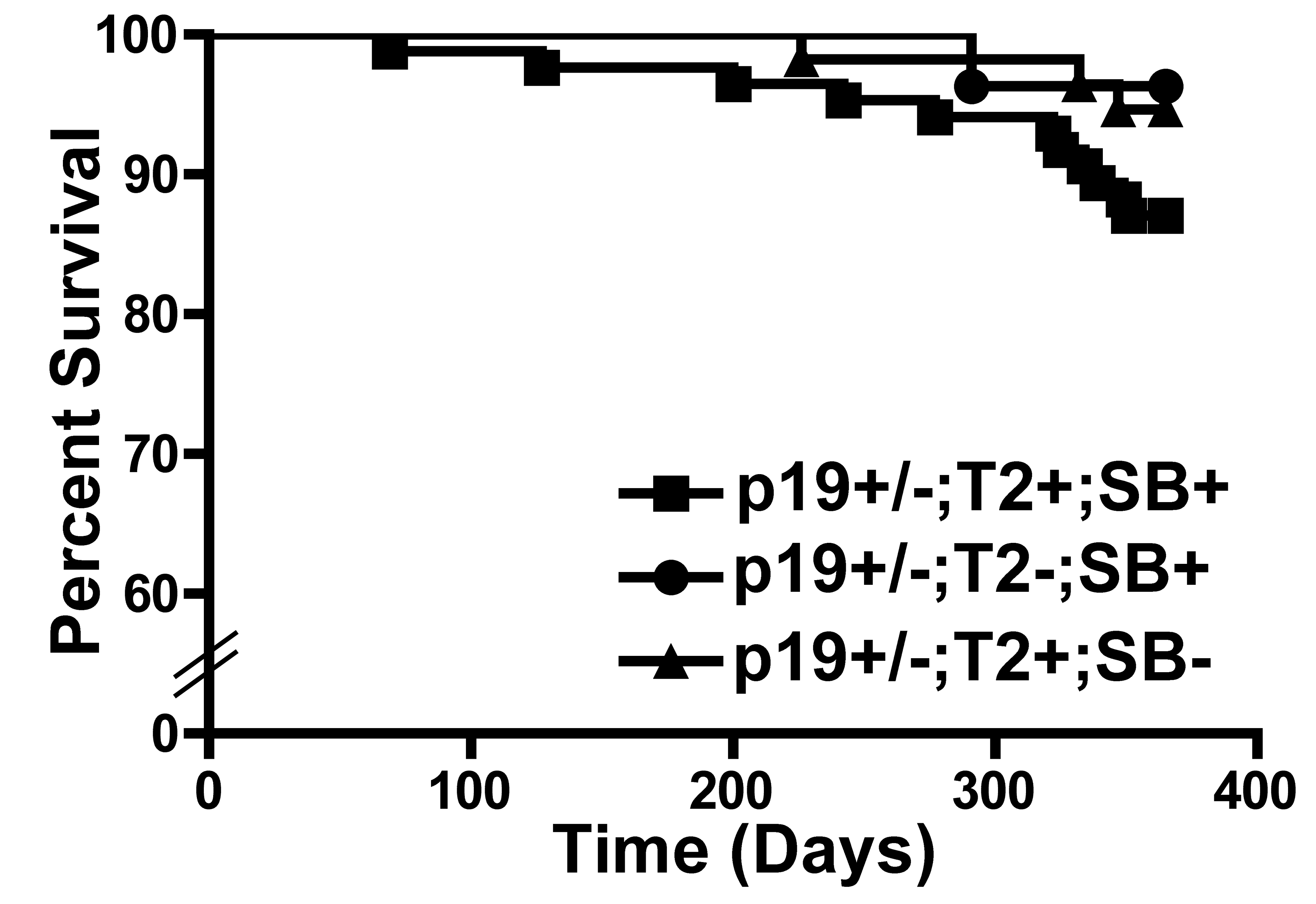

Supplement: Figure S2 — Transposon mobilization by GFAP-SB11 does not accelerate time to morbidity in p19Arf+/− mice. Kaplan Meier survival curve showing time to morbidity of p19Arf+/− mice with GFAP-SB11 mobilizing transposons (p19+/−; T2+; SB+, squares) is not statistically different (p = .1772, Logrank test) than that of control p19Arf+/− mice with GFAP-SB11 only (p19+/−; T2−; SB+, circles) or transposons only (p19+/−; T2+; SB−, triangles). Data from GFAP-SB11 A and B lines as well as two different transposon lines (T2/onc LC76 and T2/onc2 HC) were combined for analysis. (TIF) [file pone.0113489.s002.tif]

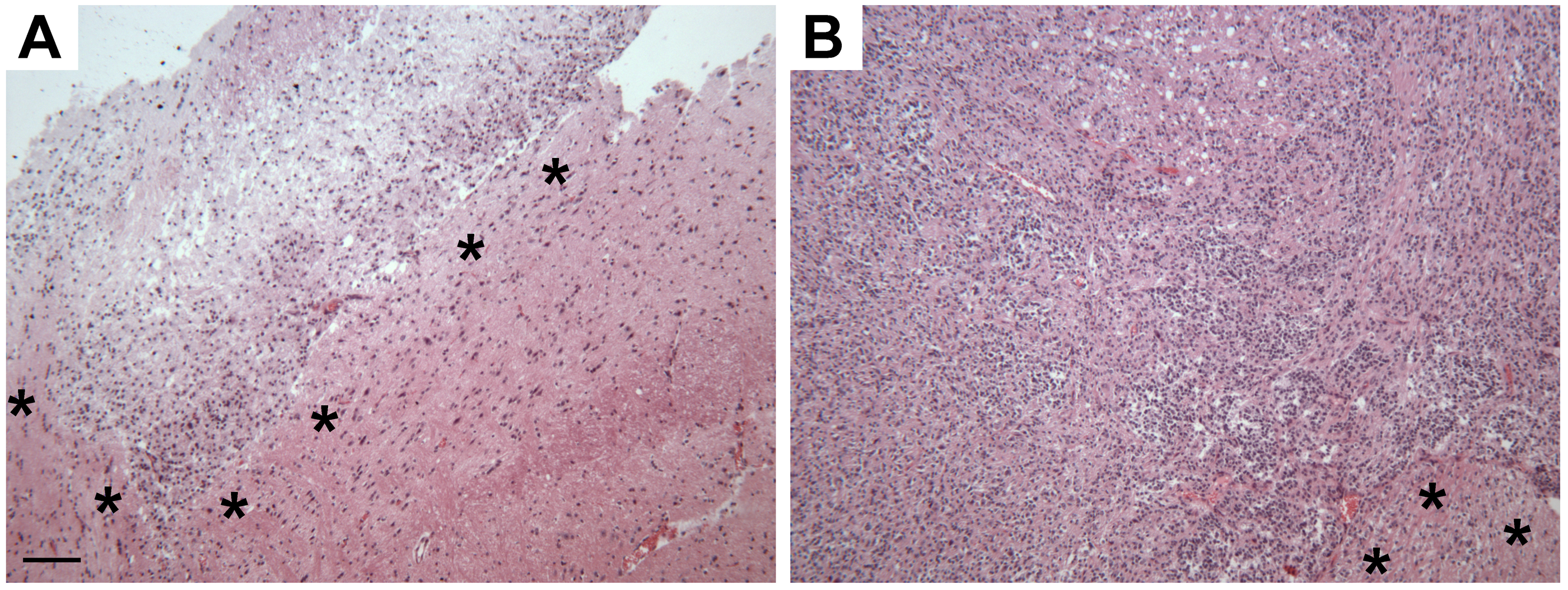

Supplement: Figure S3 — Examples of brain phenotypes from mice with mobilizing transposons. A) Hematoxylin and eosin (H&E) stained section of a low-grade glioma from a p19Arf+/−; GFAP-SB11; T2/onc mouse. B) H&E stained section of a low-grade glioma, characterized by low cellularity and no obvious mitotic activity, from a Rosa26-SB11; T2/onc mouse. Asterisks indicate ill-defined border of normal brain with tumor in each panel. Scale bar = 100 µm. (TIF) [file pone.0113489.s003.tif]

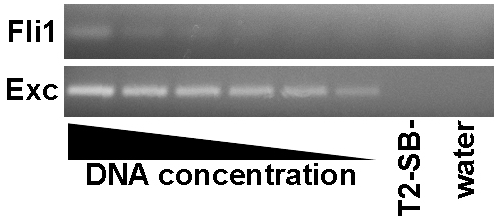

Supplement: Figure S4 — Endpoint PCR for the transposon insertion in Fli1 in the glioma in AR151. Decreasing amounts of AR151 glioma genomic DNA were used as input for the PCR. Excision PCR was used to control for genomic DNA quality, while genomic DNA from a T2/onc−; RosaSB11− (T2−SB−) mouse and water only controlled for PCR specificity. The Fli1 insertion could only be detected with high levels of input glioma genomic DNA, indicating that it is present in only a subset of cells within the tumor. (TIF) [file pone.0113489.s004.tif]
